# Further development and evaluation of a questionnaire targeting person-centred outpatient care for persons with long-term conditions

**DOI:** 10.1186/s41687-023-00623-6

**Published:** 2023-08-23

**Authors:** Fredrik Gasser, Sidona-Valentina Bala, Albert Westergren, Joakim Ekstrand, Peter Hagell

**Affiliations:** 1 The Pro-CARE Group, Faculty of Health Sciences, Kristianstad University, Kristianstad, Sweden; 2 The Research Platform for Collaboration for Health, Faculty of Health Sciences, Kristianstad University, Kristianstad, Sweden; 3 Department of Health Sciences, Faculty of Medicine, Lund University, Lund, Sweden; 4 Department of Medicine, Section of Rheumatology, Helsingborg Central Hospital, Helsingborg, Sweden

**Keywords:** Outpatient care, Patient-reported experience measure, Person-centred care, Reliability, Validity

## Abstract

**Aim:**

To further develop the Person-Centred Care instrument for outpatient care (PCCoc), evaluate its user-friendliness and content validity, and to explore its basic psychometric properties in various outpatient settings for adults with long-term conditions.

**Background:**

Person-centred care (PCC) has been identified as a key factor to provide high-quality care. However, there is still a lack of instruments that are based on a clearly defined framework for PCC for persons with long-term conditions in an outpatient context. The PCCoc is a patient-reported experience measure under development aiming to fill this gap.

**Methods:**

First, the 35-item PCCoc was reviewed and further developed in collaboration with a user-council. Second, the revised 36-item PCCoc was tested among persons receiving outpatient care for various long-term conditions. A total of 179 persons with long-term conditions from four different specialties participated in the study. User-friendliness and content validity were assessed through structured interviews and relevance ratings of each item. Content validity index (CVI) for individual items (I-CVI) and for the overall scale (S-CVI) were calculated, and basic psychometric properties of the PCCoc using classical test theory were explored.

**Results:**

It took a median of 8 min for participants to complete the PCCoc. The majority found items easy to understand, response categories distinct and that no important areas were missing. Results from the CVI analyses suggested that participants found the content of the PCCoc relevant (I-CVI range 0.82-1, S-CVI = 0.95). All psychometric properties examined were satisfactory (e.g., item-total correlations, 0.45–0.75; Cronbach’s alpha, 0.96; test-retest stability, 0.83).

**Conclusion:**

The PCCoc was considered user-friendly and relevant by the intended users, and its psychometric properties were satisfactory. This implies that the PCCoc can be a valuable instrument for evaluating and developing PCC in outpatient care for persons with long-term conditions. However, further studies of the PCCoc are needed to establish its measurement properties in various outpatient settings.

## Key points for decision makers

The Person-Centred Care instrument for outpatient care (PCCoc) is a generic questionnaire intended to measure patient-perceived levels of PCC in outpatient care for persons with long-term conditions.

The PCCoc was considered user-friendly and relevant (overall content validity index, 0.95) by persons receiving outpatient care for various long-term conditions, and exhibited good psychometric properties.

The PCCoc can be a valuable tool for evaluating and developing PCC in outpatient care for persons with long-term conditions.

## Introduction

In 2015, the World health organization (WHO) proposed a global strategy for people centred health services that introduced a paradigm shift in the way that health service is provided. According to this strategy, health care should respect the objectives, needs and participation of the individual person. Person-centred care (PCC) is emphasized as a core competency of health workers in this endeavour to change and improve the health care system [1]. This is particularly important for persons with long-term conditions; long-term conditions may be defined as conditions that currently are not curable but are controlled by medication and/or other interventions [2]. Furthermore, persons with long-term conditions are likely to need long-term contacts with health care, primarily in outpatient care, and would benefit from a more person-centred care [3]. Therefore, there is a need to assess levels of person-centeredness in outpatient care from the patient’s perspective.

PCC is built on a holistic perspective [4] and is carried out in collaboration between the healthcare and the person in need of care [5]. It is the unique person with his/her specific characteristics, needs and knowledge that should be addressed, which also includes relatives and others who play an important role in the person’s life [6, 7]. PCC has been identified as a key factor to provide high-quality care [7]. For example, studies have found PCC to be associated with shortened hospital stay [8], improved health outcomes, well-being, as well as cost-effectiveness [9–15] compared to regular care.

Even if health care professionals (HCPs) to a large extent consider PCC as important for good care, it is unclear to what extent they deliver PCC systematically and continuously [5, 16]. In order to assess the degree of PCC within units, promote the implementation of PCC and enable comparisons of interventions, there is a need to develop robust methods for evaluating PCC that are usable in various outpatient care clinics [17, 18]. To this end, patient-reported experience measures (PREMs) can be considered instrumental. PREMs aim at gathering information regarding patients’ experiences of their care which make them a vital part of gaining insight into how patients perceive the quality of care as well as in service improvement [19, 20]. Although there have been positive developments aimed at quantifying PCC from a patient perspective [21, 22], there is still a lack of instruments that are based on a clearly defined framework for PCC [23, 24] and that are directed to an outpatient context [17, 23, 25], which is the primary point of care for persons with long-term conditions.

Based on qualitative patient interviews and theoretical considerations Bala et al. [26] developed a framework that conceptualizes PCC in nurse-led rheumatological outpatient care. The framework defines PCC on a continuum from lower to higher degrees of PCC, i.e., from personalization via shared decision-making to empowerment, with communication and social environment running throughout the continuum. Based on this framework, a 24-item instrument was developed called the Person-Centred Care instrument for outpatient care in rheumatology (PCCoc/rheum), a PREM that aims to capture the degree of perceived PCC from a patient perspective [26, 27]. Evaluation of the PCCoc/rheum with persons in nurse-led rheumatological outpatient care showed high relevance and content validity [26], and testing of its measurement properties also showed promising results [27]. However, relatively large ceilings effects (about 26%) indicated that the experience of higher degrees of PCC cannot be fully captured by the instrument. Since then, modifications have been initiated with the aim to develop a generic version of the instrument applicable to persons with a long-term condition regardless of diagnosis and not only to nurse-led clinics. This generic version is called the Person-Centered Care instrument for outpatient care (PCCoc), and an initial 35-item version of the generic PCCoc has been tested in a neurological context with promising result. For example, items were generally found easy to understand, relevant and without important missing areas [28]. Here we describe its further development and assessment in diverse outpatient settings for persons with long-term conditions receiving care from different categories of HCPs.

## Aim

To further develop the PCCoc, evaluate its user-friendliness and content validity, and to explore its basic psychometric properties in various outpatient settings for adults with long-term conditions.

## Methods

### Overview

First, the 35-item PCCoc [28] was reviewed and further developed in collaboration with a user-council. Second, the revised PCCoc was tested among persons receiving outpatient care for long-term psychiatric, cardiological, rheumatological and neurological conditions. The testing was conducted in two sequentially recruited independent samples: in sample 1 we assessed user-friendliness and content validity, and in sample 2 we conducted initial testing of basic psychometric properties. Both samples received the same version of the PCCoc.

### Questionnaire development

Based on the underpinning conceptual framework [26] and previous experiences [28] the research group modified the PCCoc in collaboration with a user-council consisting of five persons (three men and two women) with long-term psychiatric, rheumatological or neurological disorders. The user-council was recruited through relevant patient organizations. The questionnaire modification process involved review of available items regarding wording, meaning and relevance, as well as consideration of the potential need for new items. This process resulted in a 36-item version (revised wording of five items and addition of one new item) that was tested in this study. For each item, respondents are instructed to indicate their experiences during the past year according to four ordered response categories (0 = completely disagree to 3 = completely agree), yielding a raw score that can range between 0 and 108 (higher scores = greater degree of perceived PCC).

### Setting

Data collection was conducted over a period of eight months (September 2021 - May 2022) in two medium-sized Swedish hospitals. Outpatient clinics in four different medical specialties (psychiatry, cardiology, rheumatology, and neurology) were included to cover a variety of persons with long-term disorders. The cardiology clinic was co-located with the inpatient ward, whereas the rest were separate outpatient units.

### Samples

Sample 1: We aimed for a sample of about 80 persons (about 20 per specialty). Inclusion criteria were adults (≥ 18 years old) with a long-term condition, who had had a minimum of three contacts (at least two physical visits) with their respective outpatient clinics during the past year. Participants should be able to speak, read and understand Swedish, be able to complete a questionnaire, and participate in an interview (as determined by their attending HCP).

Sample 2: We aimed for a sample of about 120 persons (about 30 per specialty). Inclusion criteria were as above except for the ability to participate in an interview.

Purposive sampling was employed in both samples to achieve variation in age, sex, and disease duration.

### Data collection and procedures

First, HCPs at the respective outpatient clinics were informed about the purpose and procedure of the study by members of the research team (FG, PH and SVB). Participants in both samples were then recruited by the HCPs, who provided oral and written information about the study and collected written informed consent.

Sample 1: Outpatient clinic HCPs scheduled a face-to-face interview between the person receiving care and a member of the research group, typically in connection to a planned clinic visit. Prior to the interviews, the interviewers met to review the interview procedure to ensure consistency. Before the interview, each participant completed the 36-item PCCoc individually in the presence of the interviewer who noted any signs of difficulty in responding (which were followed up during the subsequent interview) and the time taken to complete the questionnaire as an indicator of response burden [29]. This was followed by a structured interview [30] to identify any difficulties regarding the interpretation and understanding of questionnaire instructions, items and response categories, whether anything important was missing, and if the time taken to complete the questionnaire was considered acceptable. All participants’ comments were noted verbatim by the interviewer and reviewed for accuracy together with the interviewees at the end of the interview. In addition, respondents were asked about their diagnosis, how long they had received care at the outpatient clinic, the approximate number of clinic visits during the past year, and which HCP category/-ies they received care from. They were also asked if they had been thinking of a particular HCP category or their care as a whole, and approximately what time interval they had been thinking of when responding to the PCCoc. Following the interview, background data were collected regarding age, gender, country of birth, living situation, education, disease duration, perceived health (Excellent; Very good; Good; Fair; Poor; [31]) perceived disease severity (Mild; Moderate; Severe; [32]), and how they rated their ability to manage activities of daily living (No difficulties; Some difficulties; Moderate difficulties; High levels of difficulties; Extreme difficulties; [33]).

Finally, content validity was assessed from the perspective of the participants, where they were asked to indicate how relevant each PCCoc item was according to their experience using four ordered response categories (1 = Not at all relevant; 2 = Not particularly relevant; 3 = Quite relevant; 4 = Very relevant).

Sample 2: Outpatient clinic HCPs provided participants with two copies of the PCCoc, a background questionnaire (see above) and two pre-paid response envelopes. Participants were instructed to complete the questionnaire independently approximately two weeks apart.

### Data analysis

Interview data were analysed descriptively, and respondent comments were summarized based on discussions within the research group and the user-council. Content validity was assessed by the Content Validity Index (CVI) [34, 35]. First, respondents’ relevance ratings were dichotomized as relevant (responses Quite and Very relevant) or not relevant (responses Not particularly and Not at all relevant). The CVI for each item (I-CVI) was calculated as the proportion of participants rating the item as relevant divided by the total number of participants, and CVI for the whole scale (S-CVI) was expressed as the average of I-CVI across all participants. Both indices can take a value between 0 and 1 (1 = better). It has been suggested that I-CVI values ≥ 0.78 and S-CVI values ≥ 0.9 represent excellent content validity [35].

The psychometric properties of the PCCoc were explored according to classical test theory [36–38] using data from sample 2. Data completeness was assessed by the percentage of missing item data, which should be < 10% [37]. Scaling assumptions regarding the legitimacy of summing item scores into a total score assume that each item should contribute sufficiently to the total score and items should represent a common variable, which is considered supported if corrected item-total correlations exceed 0.3–0.4 [36, 38]. Floor- and ceiling effects are the proportions of respondents with the lowest (floor) and highest (ceiling) possible total scores, respectively. Up to 15–20% floor/ceiling effects are typically considered acceptable [36]. Score homogeneity was assessed by the average inter-item correlation (which should be > 0.3) [36]. Internal consistency reliability was estimated by Cronbach’s coefficient alpha. The influence on alpha when deleting each item was also examined; an increased coefficient following item deletion may suggest issues regarding, e.g., construct conceptualization or multidimensionality. In addition, test–retest stability between total scores from time 1 (T1) and time 2 (T2) was examined by a two-way mixed intra-class correlation (ICC) with absolute agreement. Reliability coefficients should exceed 0.70 and preferably 0.80 [37, 38].

All statistical analyses were performed using IBM SPSS version 28.0.1.0 (IBM Corp., Armonk, NY, USA) and Microsoft Excel (version 2208 for Microsoft 365).

## Results

A total of 179 persons with long-term conditions from four different specialties participated in the study: psychiatry (n = 46), cardiology (n = 39), rheumatology (n = 50), and neurology (n = 44). The sociodemographic and clinical characteristics of the participants are summarized in Table 1.

**Table 1:** Sociodemographic and clinical characteristics of the sample

Characteristics	Sample 1 n = 78	Sample 2 n = 101
**Speciality**, *n* (%)		
Psychiatry	20 (26)	26 (26)
Cardiology	20 (26)	19 (19)
Rheumatology	20 (26)	30 (30)
Neurology	18 (23)	26 (26)
**Age**^a^, mean (SD)	57.8 (16.2)	54.3 (17.3)
**Sex**, *n* (%)		
Woman	37 (47)	53 (52)
**Living situation**, *n* (%)		
Married/Partner	48 (62)	67 (66)
Living alone	24 (31)	26 (26)
Other	6 (8)	8 (8)
**Country of birth**, *n* (%)		
Sweden	70 (90)	88 (87)
Other	8 (10)	13 (13)
**Educational level**, *n* (%)		
Comprehensive school (9 years)	22 (28)	22 (22)
Upper secondary school (11–13 years)	32 (41)	38 (38)
University	24 (31)	41 (41)
**Main occupation**^b^, *n* (%)		
Employed	20 (26)	34 (34)
Retired	34 (45)	33 (33)
Sickness benefit	10 (13)	18 (18)
Other^e^	12 (16)	14 (14)
**General perceived health**^a^, *n (%)*		
Bad	7 (9)	7 (7)
Fair	27 (35)	28 (28)
Good	21 (27)	29 (29)
Very good	20 (26)	30 (30)
Excellent	2 (3)	7 (7)
**Perceived disease severity**^c^, *n (%)*		
Mild	24 (31)	35 (35)
Moderate	37 (47)	48 (48)
Severe	17 (22)	17 (17)
**Daily activity**^d^, *n (%)*		
No difficulties	24 (31)	34 (35)
Some difficulties	38 (49)	49 (51)
Moderate difficulties	13 (17)	11 (11)
High levels of difficulties	3 (4)	2 (2)
**Most common contacts** ^f^ *, n (%)*		
Planned visit	77 (99)	94 (93)
Visit to 1 HCP	44 (56)	40 (40)
Visit to > 1 HCP	42 (54)	60 (59)
Telephone	42 (54)	65 (64)
Emergency visit	20 (26)	32 (32)
E-mail	20 (26)	28 (28)
Education/training in groups	20 (26)	17 (17)
Individual education/training	13 (17)	12 (12)

HCP = Health care professional

^a^ n = 77, sample 1; ^b^ n = 76, sample 1; n = 99, sample 2; ^c^ n = 100, sample 2; ^d^ n = 96, sample 2

^e^Including studying and in search of work

^f^>1 answer option is possible, contacts < 10% are not reported

### Sample 1: user-friendliness and content validity

Participants (n = 78) reported a duration of their conditions of between 2.5 months up to 50 years, with a median of 7 years. The most frequent (i.e., > 5%) self-reported diagnoses were substance use disorder (18%), rheumatoid arthritis (18%), Parkinson’s disease (10%), cardiac fibrillation (10%) and cardiac infarction (9%). Participants reported a median (q1-q3) of 6 (3–16) visits in the past year and the largest proportions reported that they met nurses and physicians during the visits (Table 2).

**Table 2:** Health professionals encountered at the outpatient clinics (n = 78)

Profession	*n (%)*
Registered nurse	74 (95)
Physician	68 (87)
Physiotherapist	21 (27)
Assistant nurse	13 (17)
Occupational therapist	11 (14)
Social worker	8 (10)
Psychologist	2 (3)
Other^a^	8 (10)

^a^ Receptionist (n = 5); Peer support (n = 1); Dietician (n = 1); Speech therapist (n = 1)

The mean (SD) time taken to complete the PCCoc was 8.6 (3.8) minutes [median (q1-q3; min-max), 8 (5.8–10; 3–20) minutes], which was considered acceptable by all participants. Eighty-one per cent (n = 63) of participants reported that they thought of their care as a whole when they responded to the PCCoc, the rest thought about their nurses (n = 11; 14%), physicians (n = 3; 4%) and 1% (n = 1) thought of social workers. A majority (n = 60; 78%) were thinking of the past year when they responded to the PCCoc, and the rest thought of a time interval that ranged between 1.5 and 10 years.

Responses from interviews are summarized in Table 3. The majority (74–92%) found the design appealing, instructions simple and clear, items easy to understand and response categories easy to use. Some participants (n = 14; 18%) indicated that they thought something was missing in the PCCoc (psychiatry, n = 3; cardiology, n = 8; rheumatology, n = 0; neurology, n = 3). However, a review of aspects mentioned as missing revealed that they either related to a domain beyond the scope of the PCCoc (e.g., inpatient care) or already were covered by other items in the PCCoc.

**Table 3:** Responses from the structured interview

Interview questions	Total n = 78 (%)	Psychiatry n = 20 (%)	Cardiology n = 20 (%)	Rheumatology n = 20 (%)	Neurology n = 18 (%)
**Appealing and clear design**	72 (92)	17 (85)	18 (90)	20 (100)	17 (94)
**Simple and clear instructions**	65 (100) ^a^	15 (100)	13 (100)	20 (100)	17 (100)
**Items easy to understand**	58 (74)	14 (70)	13 (65)	19 (95)	12 (67)
**Clear and easily distinguishable response categories**	72 (92)	19 (95)	18 (90)	18 (90)	17 (94)
**Response categories easy to use**	69 (89)	17 (85)	16 (80)	20 (100)	16 (89)
**Something missing**	14 (18)	3 (15)	8 (40)	0	3 (17)

^a^n=77; n (%) of those that read the instructions

Participants’ comments related to five specific items. Twelve participants expressed concerns with items 15 (*I am given the opportunity to involve the person(s) close to me in my care*) and 25 (*The person(s) close to me receive the support they need to participate in my care*). Two issues emerged: The first was pandemic related, as the healthcare system during the past year largely did not allow relatives to participate during outpatient visits; one of the participants stated “In what way could I involve my relatives? They were not allowed to come because of the pandemic”. Secondly, participants either did not want to involve their relatives or expressed that it was relevant at the beginning of their condition, but the need had subsequently subsided. Some participants who expressed difficulty in answering the question wanted “don’t know” as an additional response option.

Eleven participants expressed difficulties with item 3 (*I am an equal part in the meeting with the health care staff*), since “equal part” was found challenging to interpret. For example, some found it unclear whether the item concerned equality on a personal level or in terms of knowledge. Some participants also expressed that as a patient, you are always at a certain disadvantage, which makes it difficult to answer.

Five participants expressed difficulties with item 1 (*The care environment is inviting for me*), in that they found it difficult to interpret the meaning of “care environment”, i.e., whether it concerned the physical environment or related more to how they were approached and treated by the staff. Some also expressed that no care environment is inviting.

Five participants raised thoughts regarding item 32 (*A written plan for my care is established together with me*). A pervasive trend was that participants expressed uncertainty about if a written plan for their care had been established at all, but still chose the response option “agree” or “strongly agree”. As one of the participants stated, “A written plan has not been drawn up, I marked “strongly agree” but I don’t know”.

Results from the CVI analyses suggested that participants found the content of the PCCoc relevant (Table 4). In the total sample, I-CVI values ranged between 0.82 and 1 and S-CVI was 0.95. Among the different specialties I-CVI were ≥ 0.78 in all instances but two (0.74 for item 1/cardiology and item 25/psychiatry) and S-CVI ranged between 0.93 and 0.97.

**Table 4:** I-CVI and S-CVI for the total sample and for each specialty

No.	Item (abbreviated)	Total	Psych	Card	Rheum	Neuro
				**I-CVI** ^a^		
1	Inviting care environment	0.82	0.84	**0.74**	0.9	0.78
2	Undisturbed conversations	0.95	0.95	1	0.95	0.89
3	Equality in meeting	0.97	0.95	1	0.95	1
4	Confirmed as person	0.99	1	1	0.95	1
5	Opportunity to tell my story	0.96	1	0.95	0.95	0.94
6	Understanding my situation	0.93	0.89	1	0.95	0.89
7	Experiences are respected	0.97	1	1	0.95	0.94
8	Self-knowledge is considered	0.97	1	1	0.95	0.94
9	Problems are taken seriously	0.99	1	1	0.95	1
10	Needs determine care planning	0.95	0.94	0.89	0.95	1
11	Agree with HCP on what to do	0.99	1	1	0.95	1
12	Gain new knowledge	0.89	0.84	0.95	1	0.78
13	Strengthened ability to cope	0.96	1	1	0.95	0.89
14	Coordinated care	0.95	0.95	1	0.9	0.94
15	Family participation	0.89	0.79	0.95	0.9	0.94
16	Care follow-up and documentation	0.97	1	1	0.9	1
17	Care responsibility is clear	0.89	1	0.84	0.9	0.83
18	Confident HCP contacts	0.97	1	1	0.9	1
19	Sufficient time allocated	0.97	1	1	0.9	1
20	Good HCP collaboration	0.99	1	1	0.95	1
21	Information facilitating decisions	1	1	1	1	1
22	Can influence care	0.99	1	1	1	0.94
23	Personal information documented	0.97	1	1	0.95	0.94
24	Care information shared as needed	0.99	1	1	1	0.94
25	Support for family members	0.87	**0.74**	0.89	0.9	0.94
26	Active participation in care	0.93	1	1	0.9	0.83
27	Encouraged to participate	0.95	1	1	0.95	0.83
28	Involved in care	0.93	0.89	1	0.95	0.89
29	Participate in care planning	0.96	1	0.95	0.95	0.94
30	Participate in decisions on care	0.99	1	0.95	1	1
31	Participate in implementing care	0.96	1	1	0.95	0.89
32	Agreed written care plan	0.88	0.89	0.84	0.9	0.89
33	Achieve care goals	0.96	0.95	1	0.95	0.94
34	Support to achieve care goals	0.99	1	1	1	0.94
35	Own resources are utilized	0.91	0.89	0.89	0.95	0.89
36^b^	Own whishes are considered	0.99	1	1	0.95	1
	** S-CVI** ^**a**^	0.95	0.96	0.97	0.94	0.93

Psych = psychiatry; Card = cardiology; Rheum = rheumatology; Neuro = neurology; I-CVI = content validity index for item; S-CVI = content validity index for scale

^a^ Values below the suggested cut-off values for excellent content validity (i.e., I-CVI ≥ 0.78, S-CVI ≥ 0.9; [35]) are bold

^b^ New item not included in the previous 35-item version of the generic PCCoc [28]

### Psychometric properties

Data quality was acceptable with ≤ 2% missing item responses. Corrected Item-total correlations ranged between 0.45 and 0.75, thus supporting the legitimacy of summing item scores into a total score and a common underlying latent variable. There was no floor effects and a 12% ceiling effect. Score homogeneity was 0.38. Reliability estimates showed a Cronbach’s alpha of 0.96 (0.95–0.96 if item deleted), and test-retest stability was 0.83 (95% CI, 0.74–0.89).

## Discussion

This study provides support for the user-friendliness and content validity of the PCCoc from the perspective of persons with various long-term conditions in an outpatient context. We also found general support for its psychometric properties according to classical test theory.

It took an average of less than 10 min to complete the questionnaire, which has been considered acceptable in terms of respondent burden [29]. In addition, all participants considered the questionnaire completion time to be acceptable. However, it has been indicated that respondent burden of answering a questionnaire could be seen as more complex than the time used to complete it. For example, it has been argued that additional information regarding, e.g., the relevance of the content from respondents’ point of view is needed [39].

Participants were generally positive about the design and content of the PCCoc. Responses from the cardiology outpatient clinic differed somewhat from the pattern and accounted for most of those who expressed that they found something was missing in the PCCoc. A possible explanation could be that the cardiology outpatient clinic differed slightly from the others, as it is located directly adjacent to the ward where many of the participants had received inpatient care, which may have made them consider aspects of their inpatient care when evaluating the PCCoc. This is supported by responses regarding whether anything important was missing in the PCCoc, where suggestions related to aspects outside the scope of the PCCoc (e.g., aspects related to inpatient care) typically were forwarded by participants from cardiology.

A few items were reported as more difficult to understand. The comments related to these items were discussed by the research group and suggestions of rewording were made (items 1, 3, 25 and 32). No ambiguity with wording emerged in the reported difficulties with item 15, where comments were related to a reduced possibility to involve family due to the pandemic or a decreased need for family participation. Revised item wordings were discussed with and assessed by the user council, who (following some additional minor revisions) considered the revised versions to address identified ambiguities while retaining their intended meaning. Previous studies on other generic PCC related instruments have also found items considering family participation/support and written care plans to be problematic, and it has been suggested that they may not represent the latent PCC variable [21, 40]. However, both family participation/support and involvement in the establishment of a written care plan are undoubtedly important aspects in the practice of PCC [4, 5, 41]. Therefore, rather than omitting these items we revised and retained them. The modified version of the PCCoc is in need of empirical testing.

Some respondents indicated that they wanted a “don’t know” response category. While this can be understandable, there is also evidence that “don’t know” does not improve measurement and in many cases factors other than lack of opinion are involved in the use of such a category [42]. While there is a potential risk of missing responses in the absence of a “don’t know” category, there were few missing responses (≤ 2%) in this study, which argues against adding a “don’t know” category.

We found good support for the participant perceived content validity of the PCCoc as S-CVI and I-CVI values were above the suggested thresholds for excellent content validity (0.90 and 0.78, respectively) [35]. This was also the case when considering the four specialties separately, except for two instances of marginally lower I-CVI values (item 1 in cardiology and item 25 in psychiatry). This provides further support for revising the wording of these items. However, a key finding was that the content validity in all four specialities was in accordance with previous results from the PCCoc/rheum [26] and the 35-item PCCoc [28], providing support for the generic nature of the instrument. Furthermore, most respondents stated that they were not considering a specific HCP category when answering the PCCoc, which supports a second generic intention of the PCCoc, i.e., that it should be useful to evaluate the overall degree of perceived PCC and independent of the profession of the care provider. The establishment of a generic instrument enables the use of a single instrument across outpatient settings, which allows for comparisons between different settings and facilitate the development of person-centred care.

All psychometric properties examined were found satisfactory. The PCCoc also showed an acceptable ceiling effect, which was less than for the PCCoc/rheum, suggesting that revisions have improved the possibility to capture higher levels of PCC. Capturing higher levels of perceived PCC has proven difficult in other generic instruments aimed at measuring person-centred care [21, 43]. However, limiting floor- and ceiling effects are fundamental for providing measurement of sufficient precision that is sensitive to differences and responsive to changes over time [44].

### Strengths and limitations

The importance of including the target group in the validation of an instrument has been pointed out [45] and there has also been a lack of user voices in evaluations of PCC instruments [17, 46]. By having intended respondents assess relevance and including a user council in the development of the PCCoc, this study contributes to filling this gap.

The study included a variety of common outpatient care specialties to enable an evaluation of how the PCCoc is experienced by people with long-term conditions in outpatient contexts regardless of diagnosis. The distribution of respondents across specialties was relatively even in both samples, and there was a variation in their sociodemographic and clinical characteristics which may be seen as a strength given the aim of developing and evaluating a generic version of the PCCoc. However, there was less variation in terms of country of birth, which affects the possibility of evaluating the user-friendliness and relevance of the PCCoc for persons with a non-Swedish origin.

Content validity was evaluated using the CVI. While the CVI is a recommended and commonly used indicator of content validity [35, 47], it should be noted that it focuses on perceived relevance, whereas content validity can be seen as a more complex property [47, 48]. However, taking a broader perspective on content validity, result from the interviews provided support for other aspects such as comprehensibility and comprehensiveness of the PCCoc.

In sample 1 we aimed for about 20 respondents per specialty. This was based on general sample size recommendations for the CVI [34, 35] and cognitive interviews [49], which both suggest sample sizes of about 10. However, given the relative complexity of PCC and to achieve more variation in participant experiences we aimed for 20 respondents per specialty [48]. The sample size for evaluating the basic psychometric properties can be seen as somewhat small, but there is support that sample sizes down to n = 20 can produce robust reliability estimates [50]. Furthermore, since questionnaire evaluation from the respondents’ perspective is recommended before psychometric testing in larger samples [48], we aimed for a relatively modest sample size in this stage of the development and testing of the PCCoc. This also precluded testing of properties such as responsiveness and external construct validity, which will need to be addressed in future studies designed for this purpose. More advanced psychometric approaches, i.e., Rasch measurement theory are also needed to more firmly evaluate the psychometric properties of the PCCoc including e.g., its unidimensionality and correspondence with the underpinning conceptual framework.

## Conclusion

We found the PCCoc to be considered user-friendly and relevant by the intended users while also exhibiting satisfactory basic psychometric properties. Thus, the PCCoc can be a valuable instrument for evaluating and developing PCC in outpatient care for persons with long-term conditions. This is important since there has been a lack of instruments to measure the degree of perceived PCC in an outpatient context. However, further studies of the PCCoc are needed, primarily to gain a firmer and more detailed understanding of its measurement properties through the application of Rasch measurement theory.

## Data Availability

The datasets generated and/or analysed during the current study are available from the corresponding author on reasonable request.
